# Affective Regulation and Trait Anger Personalities: The Buffering Effect of the Companion Animal Bond

**DOI:** 10.3390/ejihpe14080157

**Published:** 2024-08-18

**Authors:** Vincenzo Bochicchio, Cristiano Scandurra, Pasquale Dolce, Anna Scandurra, Maria Francesca Freda, Selene Mezzalira

**Affiliations:** 1 Department of Humanities, University of Calabria, 87036 Rende, Italy; 2 Department of Humanities, University of Naples Federico II, 80133 Naples, Italy; cristiano.scandurra@unina.it (C.S.); fmfreda@unina.it (M.F.F.); selene.mezzalira@unina.it (S.M.); 3 Department of Translational Medical Sciences, University of Naples Federico II, 80131 Naples, Italy; pasquale.dolce@unina.it; 4 Department of Biology, University of Naples Federico II, 80126 Naples, Italy; anna.scandurra@unina.it

**Keywords:** affective regulation, anger, personality, companion animal bond, pets, health, psychology

## Abstract

Emotional dysregulation involving anger can have severe consequences on the individual’s psychosocial and emotional functioning. This study aimed to investigate the role that the companion animal bond and the personality dimension of trait anger play in explaining affective dysregulation. A cross-sectional online survey was administered to 365 participants. Using the PROCESS macro for SPSS, a moderated model was tested to analyze the hypothesis that affective dysregulation depends on trait anger and that the companion animal bond moderates the relationship between trait anger and affective dysregulation. The results showed that the effect of trait anger on affective dysregulation increases especially when the degree of bonding to an animal companion is low, suggesting that a strong bond to a companion animal may protect individuals with trait anger from the likelihood of experiencing affective regulation problems. The psychological, health-related, and educational implications of the current anthrozoological study include the potential of the human–animal bond in acting as a facilitator of adaptive affective regulation processes, which can reduce the levels of uncontrolled anger-related emotions and the subsequent risk of out-of-control behaviors.

## 1. Introduction

Affective regulation stands as a pivotal feature of psychological well-being, representing a fundamental component of the psychological functioning that pertains to the intricate processes through which individuals manage and modulate their emotional experiences [1]. This regulatory mechanism is critical to cognitive processes, interpersonal dynamics, and mental well-being, since it encompasses the capacity to govern emotional impulses and plays a crucial role in decision-making processes, motivation, and interpersonal relations [2]. The processes of affective regulation are particularly essential in the management of negative emotions such as anger [3], and their enhancement represents a challenge both from a clinical and psychoeducational standpoint. In the case of anger management, for instance, it can be highly beneficial to identify and propose tactics aimed at reinforcing affective regulation strategies, especially in individuals predisposed to chronic angry reactions and behaviors (trait anger), which may negatively impact their daily lives and relationships [4]. The scientific literature has highlighted that the relationship with companion animals can have a significant effect on humans’ affective regulation processes, although the mechanisms of this process remain unclear [5]. This study aims to elucidate the aspects that mainly influence the improvement of affective regulation processes in individuals with trait anger. In the following paragraphs, we will delve into the construct of trait anger and its outcomes in terms of relationships and mental health, using the framework of affective neuroscience. Subsequently, we will explore the construct of affective regulation, focusing on the implications that the relationship with companion animals has in managing emotions, particularly negative ones such as anger. We will then present the current study, which aims to investigate the effect that the animal–human bond has on individuals with trait anger.

### 1.1. Trait Anger and State Anger from the Perspective of Affective Neuroscience

Anger has been traditionally subdivided into state and trait anger. State anger is a transitory condition consisting in an acutely produced emotional and physiological reaction ranging from slight irritation to violent fury and rage [6]. By contrast, trait anger refers to a personality dimension involving the individual’s chronic tendency to experience anger with higher frequency, intensity, and duration than other individuals who are not characterized by trait anger [7].

In the field of affective neuroscience, Davis and Panksepp [8] developed a personality theory that focuses on trait affects, which emphasizes how trait affects, including trait anger, emerge as complex neuroendocrine systems resulting from evolutionary adaptation processes shared by humans and all mammals. In this perspective, trait anger is conceived as an affective system capable of influencing behavior and cognition, and is measurable through the Affective Neuroscience Personality Scale (ANPS) [9,10]. By categorizing six major emotions, the ANPS detected three basic positive emotions, which refer to general Positive Affectivity and include Playfulness, Seeking, and Caring, and three basic negative emotions, which are associated with Negative Affectivity and refer to Fear, Anger, and Sadness [9].

The ANPS defined (trait) anger as “feeling hotheaded, being easily irritated and frustrated, experiencing anger verbally or physically and remaining angry for long periods” [11] (p. 273). In the ANPS, the “anger” scale has been specifically found to correlate negatively on the Five-Factor Model (FFM) [12] with Emotional Stability and Agreeableness. It has, thus, been hypothesized that anger represents a ‘blend’ of the two Big Factors of (low) Agreeableness and Emotional Stability [13]. Furthermore, the ANPS “Anger” and “Care” scales do not correlate with one another, thus suggesting that the two affective systems are independent (e.g., a person can manifest caring and angry behaviors at the same time [9]). Finally, neuroscientific studies found an inverse correlation between the “anger” dimension and the left amygdala volume [14], thus yielding biological evidence as to the relevance of dopaminergic signal transduction for the dimension of anger as a personality trait. 

Since individual emotion-based differences might represent the evolutionary oldest part of human personality, human core emotions are critical to discriminate between different personalities, and various linkages have been detected between (maladaptive) emotions and various forms of psychopathology [15]. The clinical relevance of the ANPS is extremely significant, and its impact in the field of psychopathological and psychiatric sciences over the past twenty years has been extensive. Indeed, a vast array of studies and research have utilized the constructs of affective neuroscience and the ANPS to understand the emotional foundations of numerous psychological and psychiatric disorders [16].

### 1.2. Trait Anger, Emotional Dysregulation, and the Companion Animal Bond

Emotional dysregulation involving anger can have severe consequences on the individual’s psychosocial and emotional functioning [17]. Elevated levels of trait anger are associated with difficulties in emotional regulation, which might lead to aggressive behaviors, negative mental health outcomes, and interpersonal problems [18]. In a study on mother–infant relationships, Morris et al. [19]’s findings revealed that the mother’s use of attention refocusing and joint mother–child cognitive reframing led to a lower intensity of expressed anger and sadness in the child. Ersan [20] also found that emotional regulation mediated the relationship between anger and aggression in children. In turn, Shorey et al. [21] showed that difficulties in emotional regulation and trait anger were associated with an increased perpetration of psychological aggression toward women in intimate relationships, whereby trait anger mediated the link between emotional regulation and psychological aggression. Using a measure of implicit evaluation of emotional regulation, Mauss et al. [22] revealed that the implicit positive evaluation of emotional regulation was associated with a successful, automatic, and physiologically adaptive downregulation of anger.

Anger has been proposed to be possibly regulated through a variety of techniques [23]: traditionally, cognitive–behavioral therapy (CBT) approaches posited that *reappraising* the anger-provoking situation downregulates anger by modifying negative thoughts [24]; according to the acceptance and commitment therapy (ACT) framework, *accepting* emotions like anger is a response-focused strategy that focuses on changing the process through which people interact with their emotions [25], whereas *suppressing* anger involves either the inhibition of emotion-related expressive behavior or the downregulation of the internal experience of negative affect [26].

Recently, some research studies have focused on the impact that the bond with pets has on the management of affect and emotional regulation. Indeed, in addition to being associated with attachment styles, transitional object dynamics, and self-awareness and mentalization processes, interspecific relationships have been shown to have various effects on individuals’ well-being also through affective regulation capacities [27]. Companion animals are part of social object relationships possibly fostering the individual’s socioemotional development, in that they can represent “social catalysts” [28] which facilitate human relationships and increase prosocial behavior [29]. Accordingly, companion animals can have a positive impact on the development of socio-emotional competencies such as empathy and prosocial concern [30]. Moreover, the animal–human bond can provide the person with the opportunity to establish a secure attachment relationship [31], which is known to be associated with better emotional regulation capacities. Overall, companion animals can have a positive impact on individuals who own and interact with them from both a psychological (e.g., through mood enhancement and stress and anxiety reduction) and physiological perspective (e.g., through the reduction in cortisol levels, heart rate, and blood pressure) [32,33]. Most importantly, it has been argued that companion animals can aid in the development of one’s capacity for emotional regulation [34].

On the dark side of the human–animal bond, cruel behaviors toward animals seem to be associated with unresolved trauma, insecure attachment, and difficulties in affective and emotional regulation, which might overall lead to psychopathology [35]. Within an “emotion regulation framework” [36], animal abuse can be viewed in its strict association with emotional attachment, empathic deficits, and interpersonal violence. Cruel behavior toward animals, which is often associated with antisocial attitudes, can indicate problematic attachment styles linked to emotional dysregulation, scarce empathy, and behavioral control deficits [37]. As Parfitt and Alleyne [36] stated, “animal abuse is an outcome of poor emotion regulation” (p. 62). Ultimately, even though mixed findings exist as to the evaluation of the positive or negative effects of pets on human beings [5], the person’s behavior toward their companion animal is associated with psychological variables, such as emotion (dys)regulation and different types of mental health outcomes and well-being [38].

### 1.3. The Current Study

**Figure 1:**
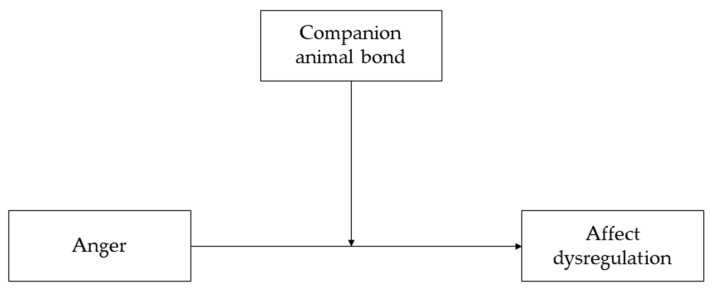
The hypothesized moderation model.

The current study aimed to investigate the role of trait anger and the companion animal bond in explaining individuals’ affective dysregulation. For this purpose, based on the previously highlighted relationship between trait anger and affective regulation, we first hypothesized that trait anger could be positively associated with affective dysregulation (Hypothesis 1). Given the known role that the bond with a companion animal can play in improving the management of affects, we expected that a stronger companion animal bond would reduce the effects of trait anger on affective dysregulation and, thus, moderate this relationship (Hypothesis 2). The hypothesized moderation model is shown in Figure 1.

Finally, we decided to include as companion animals only cats and dogs because they represent the most frequently owned pets by individuals coming from various Western countries such as Italy, which is the context of the current study. Furthermore, cats and dogs are also the most investigated pets in the scientific research on the human–animal bond, as shown in the literature review previously outlined.

## 2. Materials and Methods

### 2.1. Procedures

A cross-sectional survey was administered to a community sample recruited via the Google Forms survey software between October 2022 and February 2023. Participants were recruited on main social networks (e.g., Facebook), and those interested were asked to circulate the survey among their personal contacts, activating a snowball sampling recruitment procedure. This study was conducted in collaboration with the ENPA (Ente Nazionale Protezione Animali), the most prominent Italian national association for animal protection, which disseminated the survey through its social networks, engaging its network of activists and volunteers who work on the adoption of stray animals from shelters.

The participants were informed about the aims, benefits, and potential risks of this study and were introduced to the researchers. Informed consent was provided on the first survey page, and the participants were asked to give their consent before proceeding with the study. To avoid incomplete data, all questions were mandatory, although the participants had the option to exit the survey at any time.

This study was approved by the ethical committee of the University of Calabria (protocol number: 0048012; date of approval: 21 June 2022), developed in compliance with the EU General Data Protection Regulation, and designed in accordance with the Declaration of Helsinki.

### 2.2. Participants and Sample Size

Participants were considered eligible if they satisfied the following criteria: (1) were at least 18 years old (i.e., the Italian age of consent); (2) had lived in Italy for at least 10 years; (3) spoke the Italian language; and (4) had a dog, a cat, or both.

A statistical power analysis for the sample size determination was conducted using the G*Power software (version 3.1.9.7). Given a small effect size (*f*^2^) of 0.03, a significance level (*α*) of 0.05, a power of 0.95, and four predictors (anger, companion animal bond, age, and gender) in the multiple linear regression analyses with affective dysregulation as the dependent variable, a required sample size of *n* = 353 was identified. The survey closed after 365 participants had completed the questionnaires. Overall, 58 of the participants were men and 307 were women. The age of the participants ranged from 19 to 74 years (*M* = 37.40, *SD* = 12.03). In addition, 47.9% (*n* = 175) only had one dog, 30.4% (*n* = 111) only had one cat, and 21.6% (*n* = 79) had both.

### 2.3. Measures

#### 2.3.1. Socio-Demographic Information

The socio-demographic information collected included gender (man, woman, other), age, and current ownership of a cat, a dog, or both.

#### 2.3.2. Trait Anger

To measure trait anger, the subscale “Anger” of the ANPS [10] was used. The ANPS is based on the ethological literature and was chosen for our study given its anthrozoological nature. For the purposes of the current study, the brief form of the ANPS [39] was used in its Italian validation [40]. The short version of the ANPS is a 33-item scale on a 4-point Likert scale ranging from 1 (“strongly disagree”) to 4 (“strongly disagree”). Example items are the following: “When I am frustrated, I usually get angry” or “When someone makes me angry, I tend to remain fired up for a long time”. The McDonald’s omega coefficient in the current study was 0.71.

#### 2.3.3. Companion Animal Bond

To measure the individual characteristics of the companion animal bond, the *Companion Bonding Animal Scale* [41,42] was used to ask for the type of interaction between humans and pets. The scale, which comprises 8 items on a 5-point Likert scale ranging from 1 (“never”) to 5 (“always”), was translated into Italian using the back-translation procedure [43]. Example items are “How often were you responsible for your companion animal’s care?” or “How often did you feel that you had a close relationship with your companion animal?”. The McDonald’s omega coefficient in the current study was 0.76.

#### 2.3.4. Affective Dysregulation

To measure affective dysregulation, the *Difficulties in Emotion Regulation Scale* [2] was used in its Italian validation [44]. The scale comprises 36 items on a 5-point Likert scale ranging from 1 (“almost never”) to 5 (“almost always”). Example items are “When I’m upset, I feel out of control” or “I am confused about how I feel”. The McDonald’s omega coefficient in the current study was 0.95.

### 2.4. Statistical Analyses

The statistical analyses were carried out using IBM SPSS Statistics (Version 26). All statistical tests were conducted with a significance level of 0.05.

Correlations between variables were evaluated using the Pearson coefficient. For the quantitative variables, the mean differences between the participants who only have a dog, those who only have a cat, and those who have both were tested using an analysis of variance (ANOVA).

A hierarchical multiple regression analysis was also conducted to test the hypothesis that affective dysregulation depends on trait anger and that the companion animal bond in particular would moderate the relationship between trait anger and affective dysregulation. In the first two steps, covariates, trait anger, and the companion animal bond were included as independent variables. Then, an interaction term between trait anger and the companion animal bond was created by centering both variables and including the interaction in the third step of the regression model to test whether this interaction added explained variance to the model.

Finally, the conditional effect of trait anger on affective dysregulation at different levels of the companion animal bond (−1 *SD*, *M*, +1 *SD*) was assessed using the PROCESS Macro for SPSS, and Model 1 was applied with 10,000 bias-corrected bootstrap samples [45]. This analysis was controlled for age and gender.

## 3. Results

### 3.1. Descriptive Statistics and Bivariate Correlations

The means, standard deviations, and bivariate correlations between trait anger, the animal companion bond, and affective dysregulation are shown in Table 1. The results showed a positive correlation between trait anger and affective dysregulation and a negative correlation between the animal companion bond and affective dysregulation. In contrast, no significant correlations were found between trait anger and the companion animal bond.

The ANOVA revealed no statistically significant difference between the participants who only have a dog, those who only have a cat, and those who have both in terms of trait anger, the animal companion bond, and emotional dysregulation (all *ps* > 0.05). Therefore, this variable was not included in the subsequent analyses.

### 3.2. Associations between Trait Anger, Animal Companion Bond, and Affective Dysregulation

The hierarchical multiple regression analysis (Table 2) showed that, among trait anger and the companion animal bond, only trait anger was significantly associated with increased affective dysregulation, accounting for 22.2% of the variance. Thus, the companion animal bond was not associated with affective dysregulation.

However, including the interaction term between trait anger and the companion animal bond increased the explained variance of affective dysregulation by a further 2%. In all the steps, age was a significant covariate, suggesting that younger participants were more likely to report higher levels of emotional dysregulation.

### 3.3. Conditional Effect of Trait Anger on Affective Dysregulation at Different Levels of Companion Animal Bond

**Figure 2:**
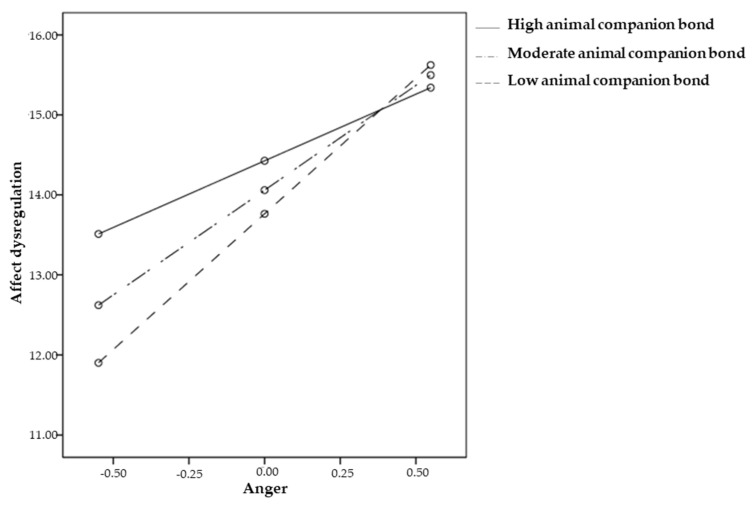
Interaction effect of trait anger by animal companion bond on affective dysregulation.

Our examination of the interaction plot showed that the effect of trait anger on affective dysregulation was significant for low (*b* = 3.39, *95%C.I.* 2.54, 4.23, *p* < 0.001), moderate (*b* = 2.62, *95%C.I.* 1.92, 3.31, *p* < 0.001), and high (*b* = 1.66, *95%C.I.* 0.65, 2.68, *p* = 0.001) levels of animal companion bond and that this effect was stronger for low levels of companion animal bond (Figure 2).

Since the effect of trait anger on affective dysregulation increases especially when the degree of bonding to an animal companion is low, it can, therefore, be argued that a stronger bond to a companion animal may protect individuals with trait anger from the likelihood of experiencing affective regulation problems. Furthermore, among the covariates, only age was found to be significant, with the younger participants showing greater problems with affective dysregulation (*b* = −0.09, *p* < 0.001).

## 4. Discussion

The current study aimed to investigate the complex associations between trait anger, the companion animal bond, and affective regulation. To this end, we first hypothesized that trait anger would be positively associated with affective dysregulation. This hypothesis was confirmed in the statistical analyses, which also indicated that a negative correlation existed between the companion animal bond and affective dysregulation. These findings suggest that trait anger might represent a specific personality facet linked to a more general affective dysregulation style, which has negative consequences on psychosocial vulnerability and interpersonal relationships [18]. Previous works in the literature showed how both trait anger and a lack of emotional awareness are associated with aggressive behavior [46], which is, in turn, associated with affective dysregulation [47].

In addition, the negative correlation between the companion animal bond and affective dysregulation indicates that one’s bond with a pet reduces the chances for poor affective regulation mechanisms in individuals owning and taking care of their pets. However, we found no significant correlations between trait anger and the companion animal bond, suggesting that trait anger might represent an independent personality factor which is likely not influenced by one’s bond with a pet.

Our results also showed that no statistically significant difference existed between the participants who only owned a dog, those who only owned a cat, and those who owned both in terms of trait anger, companion animal bond, and affective dysregulation. This suggests that the type of pet owned is not relevant in differentiating individuals in terms of levels of trait anger, the relationship with one’s pet, and affective regulation processes.

Furthermore, we found that age was a significant variable in accounting for affective dysregulation processes, with the younger participants being more likely to report higher levels of affective dysregulation. From a life span perspective, this might be explained by the different developmental trajectories that characterize younger people compared to older individuals. More specifically, these results might indicate that the challenges of the developmental tasks that younger people need to face expose them to higher chances for affective dysregulation compared to older people, as demonstrated by previous research showing how younger individuals, especially adolescents, go through psychophysical changes, leading to more significant psychopathological vulnerabilities both in terms of internalizing and externalizing problems [48], which are, in turn, linked to affective dysregulation processes [49].

As for the second hypothesis of our study, we expected that a stronger companion animal bond would reduce the effects of trait anger on affective dysregulation and, thus, moderate this relationship. The results confirmed this hypothesis, showing that the companion animal bond moderated the relationship between trait anger and affective dysregulation. More specifically, a stronger attachment to a companion animal emerged as a protective factor that could aid, at least partially, in preventing individuals with trait anger from showing affective regulation problems. Furthermore, since the younger participants showed greater problems with affective dysregulation than their counterparts, it is plausible that the bond with a pet might benefit younger people to a greater extent than older individuals in terms of the association between trait anger and affective dysregulation. However, future studies should investigate this hypothesis using more appropriate and targeted research designs and strive to recruit more age-balanced samples.

The results of the present study show how the bond with companion animals can modulate the impact that personality features such as trait anger can have on the capacity for affective regulation, consisting of the ability for both self- and co-regulating affective dynamics [50]. Taken together, affect and emotional regulation (i.e., the capacity to initiate, modulate, and cease emotional responses and emotion-based behavioral responses to achieve personal and socially significant goals [51,52]) can render adaptive interactions possible, both with other humans and with pets [53]. More specifically, our findings show that the human–animal bond can protect individuals from the impact that problematic personality characteristics such as trait anger have on affective dysregulation processes, thus aiding the person in adaptively relating to others, whether they are humans or companion animals.

The findings of the current study are in line with previous works in the literature showing how the animal–human bond has beneficial effects on individuals from both a psychological and a physiological point of view [32,54,55]. The animal–human bond has been shown to provide individuals with substantial emotional benefits (e.g., increased self-esteem and a feeling of connectedness) and is associated with increased social competencies and more positive interpersonal interactions [56]. For instance, a systematic review by Villafaina-Domínguez et al. [57] revealed that dog-based animal-assisted interventions could improve various variables, including mental health, emotional control, and empathy in male and female inmates.

The companion animal bond has also been shown to be effective in buffering the impact of stressful environments on the individual’s well-being [58]. Since stress needs to be faced with effective emotional regulation skills, we can argue that the interspecific bond with dogs and cats as companion animals, modulating the effect of trait anger on emotional dysregulation, can also be effective in reducing the individual’s perceived stress. Also, since stress and trauma are strictly interrelated, it is worth noting the promising clinical perspective regarding the human–animal bond as an effective relationship in the process of healing from trauma and the related hyper-reaction to stressful situations. More specifically, our findings can allow us to argue that the healing relationship with a pet can aid in emotional regulation capacities that foster the recovery process in traumatized individuals [59].

Notably, a good-enough relationship with a significant other is well-known to foster adaptive affective regulation processes. In the psychological literature, the quality of the relationship has also been widely shown to represent the most effective predictor of good outcomes in terms of mental health and well-being. The significant other can be embodied by various figures, which range from the primary caregiver for the infant, to the peer group during adolescence, to the partner in adult life couples, and to the companion animal throughout the stages of the life cycle. Specifically, the literature on the human–animal bond has shown that companion animals can represent significant “others” for human beings, thus functioning as friends, siblings, and even children, provided that the relationship with the pet is adaptive and not characterized by exploitation or even cruelty. Ultimately, the ancient connection with companion animals can feature as an effective way to engage with significant others and self-regulate.

Overall, our findings confirm previous works in the literature demonstrating how poor emotional regulation skills are associated with both trait anger and insufficient self-control [60]. Maladaptive patterns of emotional regulation processes, operationalized as comprising a lack of emotional awareness and difficulties in refraining from impulsive behavior, have been found to predict a broader history of violence in individual offenders [61]. Trait anger expressions are associated with a relative inability to refrain from impulsive behaviors and maladaptive affective regulation strategies [62]. As a result, both trait anger and affective dysregulation—as affective constructs—have been proposed to interact in predisposing an individual to aggressive behaviors [3].

### 4.1. Limitations

This study has some limitations. First, it is a cross-sectional study, thus not capturing the developmental impact that the relationship with a companion animal may have on affective regulation strategies. Future longitudinal studies will be necessary to understand the impact and dynamics that the bond with companion animals has on affective regulation strategies in individuals with high levels of trait anger. Another limitation is due to the gender imbalance in the sample, as, in our study, the number of women was significantly higher than men. Despite these limitations, our results provide some interesting insights into the effects that the relationship with animals can have on affective regulation strategies in individuals who may more easily adopt uncontrolled conduct and behaviors due to their personality traits. As a further limitation, we did not specifically measure aggressive behavior, but only trait anger. Even though the literature points to a link between the two, this could not be ascertained in the present study. However, it is worth noting that, to our knowledge, this is the first study on this topic, which adds value to our findings on the buffering effect of the human–animal bond on the relationship between trait anger and affective dysregulation. Nonetheless, we hope that future studies will fill this gap, also confirming the implications of the explicative hypotheses of our study.

As a final limitation, we should mention that, in the current study, we decided that an inclusion criterion was having been residing in Italy for at least 10 years. The rationale underlying this choice is that the anthrozoological literature has widely shown that the sociocultural context and the national legislation in a specific country significantly impact individuals’ relationship with animals, especially pets [63]. Specifically, in Italy, which is the context of the present study, the legislation grants pets some kind of civil rights, so that mistreatment and neglect of pets is severely punished, and the Italian Parliament recently included the protection and care of animals in the Constitution [64]. Since the cultural and legal framework providing a perception of the value of the human–animal relationship widely varies from country to country, we considered a 10-year period of residence in Italy as an effective parameter to better assess the awareness of the right to citizenship that pets have in our country, making the sample more homogeneous and not altering the characteristics of the variables analyzed. This might represent a limitation in that our study is rooted in a specific context, namely Italy, where the relationships between the investigated variables acquire significance within the sociocultural framework in which they have been investigated. Nonetheless, future research should address these relationships in countries where the legislation protecting animals and especially pets is scarce or even absent. Therefore, our inclusion criteria, albeit representing a potential limitation, let us suggest future directions for research in different sociocultural contexts, where the human–animal relationship might be influenced by different legislative frameworks.

### 4.2. Health-Related, Psychological, and Educational Implications

Based on the literature indicating the efficacy of the animal–human bond in positively aiding affective regulation processes [65], we suggest the critical relevance of interspecific relationships in providing individuals with positive opportunities to exhibit better affective regulation capacities. These results seem all the more relevant since affective dysregulation tends to be associated with animal abuse [36], which has been found to often be a preliminary behavior to antisocial behaviors related to human interpersonal violence [35]. Indeed, whereas affiliative interspecific relationships are associated with a better quality of life [29,54], negative behaviors toward pets are related to poor mental health outcomes [35,38]. Therefore, owning and caring for a pet can represent a protective factor in the face of the development of maladaptive affective regulation styles related to personality features such as trait anger.

A predisposition to uncontrolled behaviors led by angry emotional traits can also be caused by alexithymic characteristics in the individual [66] and has been found to be influenced by mentalization deficits [67], suggesting that the trajectory leading from trait anger to affective dysregulation is characterized by difficulties in recognizing and mentalizing emotions. In this regard, a recent experimental study [68] showed that the relationship with animals significantly enhanced individuals’ ability to recognize and mentalize their own emotions and the emotions of the animals with which they interacted. Therefore, the relationship with animals likely affects affective regulation capacities as it contributes to recognizing and mentalizing one’s own and others’ emotional states.

Ultimately, trait anger can be effectively counteracted by effortful control processes [66] that are, in turn, associated with affect- and self-regulation skills [69,70]. In this sense, the companion animal bond can act as a facilitator of affective regulation processes, thus reducing the levels of uncontrolled anger-related emotions and the subsequent risk of out-of-control behaviors performed by individuals with high trait anger. Indeed, in psychoeducational settings or in anger management courses targeting individuals with high trait anger, the companion animal bond might foster positive relational dynamics, thus helping the person to better build and consolidate adaptive affective regulation strategies. This seems all the more relevant in contexts (e.g., prisons) where companion animals have been shown to be successfully introduced to reduce recidivism in aggressive behaviors and improve individuals’ mental health and well-being from a psychological and educational perspective [57]. Future research should also further clarify the nature of the relational processes underlying the companion animal bond [71] to enable the development of more appropriate and individualized psychoeducational interventions with regard to the personal characteristics of the individuals involved.

## 5. Conclusions

The results of this study show how the relationship with companion animals can enhance affective regulation processes in individuals with high trait anger. These findings have numerous developmental, educational, and rehabilitative implications, suggesting that the relationship with animals could serve as a valuable avenue for rehabilitation in particularly stressful contexts for individuals struggling with anger management, or even for those who have difficulties in mentalizing their own affective states.

## Figures and Tables

**Table 1:** Correlations between trait anger, animal companion bond, and affective dysregulation.

	1	2	3	*M*	*SD*
1. Anger	−			1.32	0.55
2. Animal companion bond	0.01	−		4.63	0.46
3. Affective dysregulation	0.40 ***	−0.10 *	−	2.34	0.69

Notes: *M* = mean; and *SD* = standard deviation. * *p* < 0.05 and *** *p* < 0.001.

**Table 2:** Regressions of affective dysregulation on trait anger and companion animal bond.

	Affective Dysregulation		
	*B*(*SE*)	*β*	95% *CI*
*Step 1*			
Age	−0.10(0.02)	−0.30 ***	−0.14, −0.07
Gender (man)	1.01(0.57)	0.09	−0.11, 2.13
	*R*^2^ = 0.10; *F* = 20.23 ***		
*Step 2*			
Age	−0.09(0.02)	−0.26 ***	−0.12, −0.06
Gender (man)	0.73(0.54)	0.64	−0.32, 1.79
Trait anger	2.71(0.35)	0.36 ***	2.01, 3.41
Companion animal bond	−0.69(0.43)	−0.08	−1.55, 0.17
	*R*^2^ = 0.22; Δ*R*^2^ = 0.13; *F* = 26.98 ***		
*Step 3*			
Age	−0.09(0.02)	−0.25 ***	−0.12, −0.05
Gender (man)	0.75(0.53)	0.07	−0.29, 1.80
Trait anger	2.62(0.35)	0.34 ***	1.92, 3.31
Companion animal bond	−0.81(0.43)	−0.89	−1.66, 0.04
Interaction term	2.08(0.76)	0.13 **	0.59, 3.58
	*R*^2^ = 0.24; Δ*R*^2^ = 0.16; *F* = 23.48 ***		

Notes: *B* = non-standardized regression coefficient; *SE* = standard error; *CI* = confidence interval; *β* = standardized regression coefficient; *R*^2^ = R-square; and Δ*R*^2^ = R-square change. ** *p* < 0.01 and *** *p* < 0.001.

## Data Availability

The data supporting the conclusions of this article will be made available by the authors upon request.
